# The MaDo real-life study of dose adjustment of allergen immunotherapy liquid formulations in an indication of respiratory allergic disease: Reasons, practices, and outcomes

**DOI:** 10.3389/falgy.2022.971155

**Published:** 2022-08-09

**Authors:** Marguerite Thétis-Soulié, Maxime Hosotte, Isabelle Grozelier, Claire Baillez, Silvia Scurati, Valérie Mercier

**Affiliations:** ^1^ Private Office, Créteil, France; ^2^ Centre d’Allergologie de Gentilly, Nancy, France; ^3^ Private Office, Cholet, France; ^4^ Private Office, Perpignan, France; ^5^ Global Medical Affairs Department, Stallergenes Greer, Antony, France; ^6^ Private Office, Toulouse, France

**Keywords:** allergen immunotherapy, precision medicine, allergic rhinitis, dose adjustment, precision dosing, patients profiling

## Abstract

Sublingual allergen immunotherapy (SLIT) is a safe, effective, disease-modifying treatment for moderate-to-severe respiratory allergies. The function and responsiveness of the immune system components underlying the effects of allergen immunotherapy may vary from one patient to another. Furthermore, the severity of the symptoms of allergic disease can fluctuate over time, due to changes in environmental allergen exposure, effector cell responsiveness, and cell signaling. Hence, the allergen dose provided through SLIT can be fine-tuned to establish an optimal balance between effectiveness and tolerability. The objective of the MaDo study was to describe and understand dose adjustments of SLIT liquid formulations in France. We performed a retrospective, observational, cross-sectional, real-life study of allergists and other specialist physicians. Physicians described their patients *via* an anonymous case report form (CRF). The main patient inclusion criteria were age 5 years or over, at least one physician-confirmed IgE-driven respiratory allergy, and treatment for at least 2 years with one or more SLIT liquid preparations. A nationally representative sample of 33 specialist physicians participated in the study. The physicians' main stated reasons for dose adjustment were adverse events (according to 90.9% of the physicians), treatment effectiveness (60.6%), sensitivity to the allergen (42.4%) and other characteristics (30.3%: mainly symptom severity, type of allergen, and asthma). 392 CRFs (mean ± standard deviation patient age: 27.8 ± 17.5; under-18s: 42.1%; polyallergy: 30.9%) were analyzed. Respectively 53.6%, 25.8%, 15.3%, and 8.7% of the patients received house dust mite, grass pollen, birch pollen and cypress pollen SLIT. Dose adjustments were noted in 258 (65.8%) patients (at the start of the maintenance phase for 101 patients (39.2%) and later for 247 (95.7%)). Dose adjustment was not linked to sex, age, or the number of allergens administered. All measures of disease severity (including symptom severity noted on a 0-to-10 visual analogue scale by the physician) decreased significantly during SLIT. Notably, the mean AR symptom severity score decreased to a clinically relevant extent from 7.6 at SLIT initiation to 2.4 at last follow-up, and the mean asthma symptom severity score decreased from 5.0 to 1.3. The few differences in effectiveness between patients with vs. without dose adjustment were not major. For about one patient in five, a specialist physician decided to reduce or increase the SLIT liquid dose at the start of maintenance treatment and/or during maintenance treatment. This decision was influenced by a broad range of patient and treatment factors, mainly to improve tolerability to treatment and/or enhance effectiveness. In France, dose adjustment of SLIT liquid preparations as a function of the patient profile and/or treatment response is anchored in clinical practice. Precision dosing might optimize the overall benefit-risk profile of AIT for individual patients throughout their entire treatment course, enabling them to achieve both short- and long-term treatment goals, whilst maximizing the safety and tolerability.

## Introduction

Allergic rhinitis (AR) is the most common atopic disease. Worldwide, the estimated prevalence of AR ranges from 9% to 42%, depending on the study country and population (1–5). Moderate to severe AR has a negative impact on health, quality of life, and academic/work performance in children and adolescents (6–10). Furthermore, the presence of AR is thought to drive the “allergic march” of atopy towards the development of allergic asthma (AA), with the risk of potentially life-threatening adverse events (11, 12). Although symptomatic medications (such as antihistamines and corticosteroids) can often provide short-term relief, long-term treatment with these drugs may be poorly tolerated, unwanted, or incapable of achieving a sufficient decree of disease control (13, 14).

Allergen immunotherapy (AIT) is currently the only disease-modifying treatment for respiratory allergies (i.e., moderate-to-severe AR and/or moderate, well-controlled asthma). This safe, effective approach typically provides symptom relief, reduces symptomatic medication use, and is associated with better quality of life (15–23). Furthermore, AIT might stop (or at least slow) the “allergic march” and thus prevent the progression from AR to AA (24, 25). Both sublingual and subcutaneous AIT formulations are currently available for the most common disease-inducing aeroallergens. In sublingual allergen immunotherapy (SLIT), an allergen extract can be formulated as a sublingual tablet or as an aqueous liquid extract. Each type of formulation has advantages and disadvantages, and so the patient and his/her allergy specialist can choose the most suitable treatment option, as a function of the patient's immunologic and clinical profiles, lifestyle, and expectations. SLIT liquid formulations have the advantages of home administration, flexible composition and dosing, and thus lend themselves well to the concept of precision medicine.

Precision medicine can be defined as choosing the most appropriate treatment (with the right dose and at the right time) for an individual or a small group of patients as a function of immunologic, molecular, clinical and lifestyle-related variables (26, 27). For practical reasons, randomized, controlled trials (the “gold standard” for determining the efficacy and safety of medications) typically involve a few fixed dose levels or regimens (in Phase II dose-ranging studies) or a single dose level or regimen (in Phase III pivotal studies). The trial results provide population-level data on optimizing the risk-benefit ratio. However, some individual patients will benefit more (or less) than the population as a whole from fixed dose levels or regimens.

AIT in general and SLIT in particular lend themselves well to precision dosing, offering the potential for “optimal” treatment on an individual patient level (26, 27). Firstly, it is well known the function and responsiveness of the innate and acquired immune system components underlying the effects of SLIT are subject to interindividual and intraindividual variability (28–30). Secondly, the severity of the symptoms of allergic disease can fluctuate over time, due to the combined influence of environmental allergen exposure and changes in effector cells and cell signaling. Hence, the allergen dose provided through SLIT should be fine-tuned to establish the best possible balance between effectiveness and safety, both of which are dose dependent. Indeed, individual-level dose adjustment is sometimes required as a function of the patient's immunological, clinical and/or reactivity profiles and the presence or absence of intercurrent diseases (e.g., respiratory tract infections). Although drug manufacturers typically recommend a particular maintenance dose of SLIT (i.e., the dose validated in clinical trials), in liquid formulations the number of actuations can be modified in order to increase or decrease the administered dose of allergen. This fine-tuning of the dose of allergen fits well with the concepts of precision and personalized medicine (26, 27). Hence, the goal of the retrospective, multicenter, observational “MaDo” study was to evaluate real-life clinical practice for the dose adjustment of STALORAL® SLIT liquid formulations (Stallergenes Greer, Antony, France) in the treatment of allergic disorders of the upper and lower respiratory tract. The participating physicians filled out case report forms (CRFs) to describe the doses (whether adjusted or not) received by individual patients treated with SLIT liquid formulations, the strategies, reasons, duration and impact of dose adjustments.

## Materials and methods

### Study design and procedures

We performed a multicenter, nationwide, retrospective, longitudinal, observational study in France. Allergists and other physicians with expertise in treating allergies (*n* = 438, listed in a proprietary database) were contacted by e-mail and invited to participate in the MaDo study. Only physicians who stated that they adjusted the SLIT dose for at least some but not all their patients were included in the study (84% of the respondent physicians). The first 50 physicians who agreed to participate were invited to fill out a detailed questionnaire on their practice in general and their practice with regard to dose adjustment for patients taking SLIT. The physicians provided information on demographics, the type of practice (private practice and/or hospital practice), their specialty (family physician, pulmonologist, allergist, ENT specialist, pediatrician, etc.), their monthly active case file of patients with respiratory allergy, patients receiving AIT, and patients treated with SLIT solutions, their routine practice with regard to STALORAL® treatment protocols and dose adjustments, and their reasons for dose adjustment. Next, the physicians were asked to fill out detailed anonymized online CRFs on patients receiving a STALORAL® SLIT liquid formulation (Stallergenes Greer, Antony, France). We requested that two-third of the CRFs should describe patients with a dose adjustment, as defined below. For each case, the period of data collection ran from the initiation of STALORAL® treatment to the most last available status in the patient's medical records at the time of the report. Since this was a retrospective observational study, any pharmacovigilance incidents had already been notified to the regional pharmacovigilance center in the usual manner. However, the study protocol had reminded participating physicians about their pharmacovigilance reporting obligations.

### Patient eligibility criteria and outcome measures

The main inclusion criteria, applied to patients with dose adjustment and those without: (i) age 5 years or over, (ii) an intermittent or persistent IgE-driven respiratory allergy (moderate-to-severe AR, conjunctivitis or rhinoconjunctivitis or mild-to moderate AA), and (iii) treatment with one or more STALORAL® solutions for at least 2 years. The main non-inclusion criterion was treatment with AIT products other than STALORAL®.

As mentioned above, the study's primary objective was to evaluate patterns of dose adjustment of STALORAL® SLIT liquid formulations in real-life clinical practice in France. The study's secondary objective was to document the clinical effectiveness of STALORAL®, as assessed by the physician on their medical records. Hence, in each CRF, the physician provided detailed information on the patient's demographics, personal medical history with regard to allergy and clinical profile upon SLIT initiation: symptom description with ARIA classification for AR (15, 31, 32), GINA classification for asthma (33), and their intensity (using a Visual Analogue Scale (VAS) and the five-item PAREO^1^ nasal symptom score also known as the Lebel score or the Bousquet score (34)). The course of SLIT with STALORAL® was described: allergen extract(s) used, planned duration of treatment (over year and in total), the criteria that promoted the prescription of STALORAL® and the initially envisaged SLIT regimen for initiation and maintenance (dose level and dosing frequency). Lastly, the physician documented the evolution of the patient's condition from treatment initiation and last visit: symptom severity (VAS and PAREO scores), symptomatic medication use and overall impact on quality of life and, if applicable, provided details of the SLIT regimen after adaptation with the reasons for dose adjustment decision, and the impact of the dose adjustment.

### The study product and dose adjustments

STALORAL® SLIT solutions are indicated in the treatment of IgE-driven allergy induced by seasonal or perennial exposure to a specific aeroallergen. The treatment typically starts with a titration (up-dosing) phase during which the daily dose is increased up to 300 IR (or 100 IR for the *Alternaria* extract and [at the time of the study] the cat dander extract) over a period of up to 13 days using 10, 100 and/or 300 IR/ml concentrations, or with a 5-day titration starting directly with the 300 IR/ml concentration. The subsequent maintenance phase then comprised a recommended fixed dose (typically taken daily) of 300 IR (or 100 IR for *Alternaria* and cat dander extracts) i.e., 5 actuations per day with the 300 IR/ml (or 100 IR/ml) concentration. However, the dose level and regimen can be adjusted according to the patient tolerability to the treatment or the occurrence of intercurrent illness (e.g., respiratory tract infections). Here, dose adjustment was defined as at least one physician-prescribed change (whether temporary or permanent) in the recommended daily maintenance dose for the allergen in question.

### Sample size calculation and statistical analysis

In order to provide estimates for 50% of the case reports with a measurement precision of ±5%, we calculated that 384 exploitable observations would be required. Assuming a missing data rate of 10%, the target was set to 427 case reports. On the basis of earlier studies, we assumed that around 12% of the 438 invited physicians would agree to participate, and that 90% of these would include at least one case report (i.e., 53 active participating physicians). Hence, in order to obtain 427 case reports in total, we asked each participating physician to document between 5 and 10 patients.

The study's logistic aspects and data management were handled by a contract research organization (IQVIA, La Défense, France). Quantitative variables were reported as the mean ± standard deviation (SD), median [interquartile range (IQR)], and range. The 95% confidence interval (CI) was presented when relevant. Qualitative variables were reported as the frequency (percentage). Groups were compared using Student's test or Wilcoxon's test (for quantitative variables) and a chi-squared test (for qualitative variables). Before vs. after changes in the criteria describing the treatment benefits in the two study groups (i.e., patients with and without dose adjustment) were compared in an analysis of covariance adjusted for the variables' values before treatment initiation.

The threshold for statistical significance was set to *p* < 0.05. All statistical analyses were performed with R software (R: A language and environment for statistical computing; R Foundation for Statistical Computing, Vienna, Austria. URL https://www.r-project.org/).

### Ethics

In line with the French legislation on non-interventional observational studies that do not modify medical care, approval by an independent ethics committee was not required. The study was performed in compliance with the International Society for Pharmacoepidemiology's Guidelines for Good Pharmacoepidemiology Practices (https://www.pharmacoepi.org/resources/guidelines_08027.cfm), French and European legislation, and the tenets of the Declaration of Helsinki.

## Results

### Characteristics of the participating physicians

**Figure 1:**
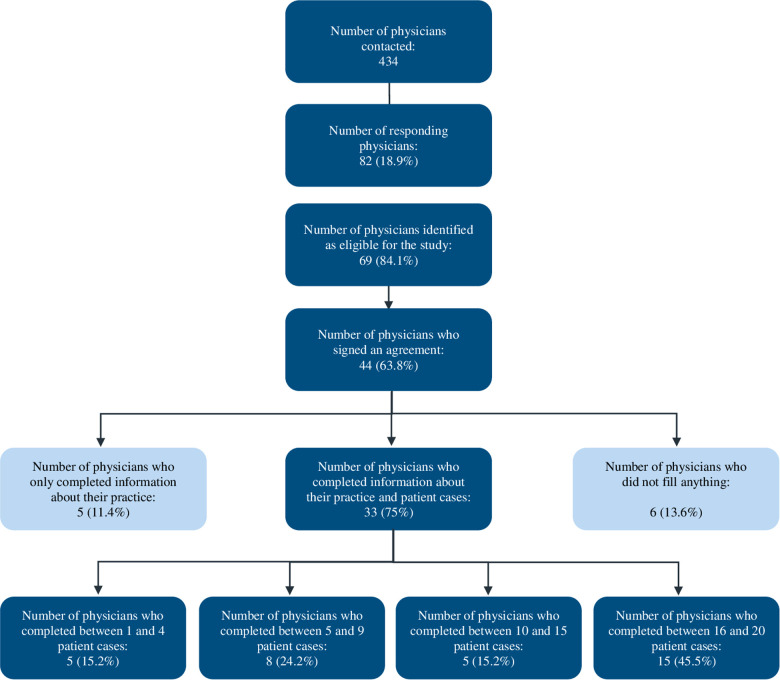
Study flow chart for physician recruitment and participation in the MaDo study.

Of the 434 physicians contacted by e-mail, 82 (18.9%) agreed to participate in the study (Figure 1). Sixty-nine of the 82 physicians (84.1%) were eligible to participate because they reported adjusting the SLIT dose for some but not all of their patients. Ultimately, 44 of these 69 physicians (63.8%) signed a study agreement, and 33 of the 44 (75%) included at least one CRF.

The characteristics of the participating physician population are summarized and compared with a reference population (433 allergists and other physicians with expertise in treating allergies in France, as listed in the OneKey® database (IQVIA, La Défense, France)) in Table 1.

**Table 1:** Characteristics of the participating physicians and a reference population of allergists and other physicians with expertise in treating allergies in France (listed in the OneKey® database from IQVIA, La Défense, France).

	Physicians participating in the MaDo study (*n* = 33)	Physicians in the reference population (*n* = 433)	*P-*value
Age, years			**0.01**
Mean ± SD	53.4 ± 9.1	58 ± 11	
Median [IQR]	56 [45–61]	61 [54–65]	
Range	37–67	0–78	
Sex			0.29
Female	15 (45.5%)	245 (56.6%)	
Male	18 (54.5%)	188 (43.4%)	
Specialty			0.059
Allergist	30 (90.9%)	273 (63%)	
Pulmonologist	2 (6.1%)	98 (22.6%)	
GP	1 (3%)	29 (6.7)	
Pediatrician	0 (0%)	25 (5.8%)	
ENT specialist	0 (0%)	6 (1.4%)	
Internal medicine	0 (0%)	2 (0.5%)	
Practice			0.17
Private practice only	25 (75.8%)	259 (59.8%)	
Private practice and hospital practice	8 (24.2%)	152 (35.1%)	

SD, standard deviation; IQR, interquartile range; GP, general practitioner; ENT, ear, nose and throat.

Most of the 33 participating physicians were allergists working in private practice. No pediatricians, ENT specialists or internal medicine specialists participated. However, the only statistically significant difference between the participating physician population and the OneKey® French reference population was age; on average, the participating physicians were younger than the physicians in the national reference population. We conclude that overall, the participating physicians constituted a nationally representative sample.

### The physicians’ practice and attitudes with regard to dose adjustment of SLIT liquid formulations

**Figure 2:**
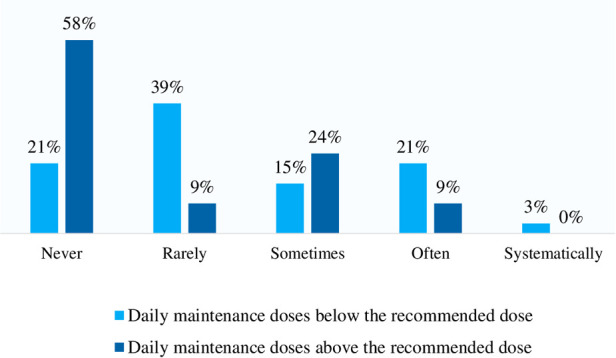
Medical practice in terms of dose adaptation at the end of the initiation phase as reported by the respondent physicians (*n* = 33).

When completing the questionnaire on their practice, the physicians were asked to state the overall frequency with which they adjusted the initial maintenance dose, right after SLIT titration, downward or upward. Downward adjustment was more frequent than upward adjustment; for example, 21% of the physicians reported often reducing the dose, whereas 9% reported often increasing the dose (Figure 2). At the start of maintenance treatment, dose reductions or increases were applied for 21.3% and 18.6% of patients, respectively.

**Figure 3:**
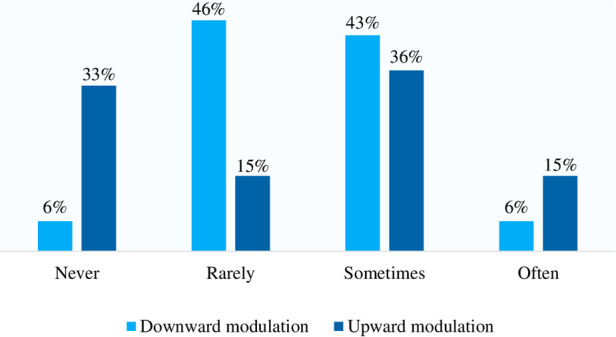
Medical practice in terms of dose adaptation during the maintenance phase as reported by the respondent physicians (*n* = 33).

With regard to the maintenance phase itself, a majority of physicians stated that they sometimes or often applied dose increases and dose reductions (Figure 3). During maintenance treatment, doses reductions or increases were performed for 13.8% and 19.0% of patients, respectively.

When asked about the most common reasons for maintenance dose adjustments, the physicians mainly mentioned the fear of occurrence of side effects (90.9%) and the effectiveness of AIT during treatment (60.6%) (Table 2). Less frequently mentioned reasons for dose adjustment were the severity of the patient's allergy, the number of clinical allergies, the particular allergen involved (mainly birch for dose downward adjustments), the presence or absence of concomitant asthma, the presence or absence of oral allergy syndrome, and the patient's age.

**Table 2:** The physicians’ stated reasons for adjusting the dose of SLIT.

Reason	Total (*n* = 33)
Occurrence or fear of adverse events	30 (90.9%)
The expected efficacy of AIT	20 (60.6%)
The patient's sensitivity	14 (42.4%)
The patient's demographic and clinical profile	10 (30.3%)
The prescription of a mixture of allergens	8 (24.2%)
The severity of the patient's allergy	8 (24.2%)
The nature of the allergen responsible for symptoms	6 (18.2%)
The presence or absence of asthma in a patient with AR	5 (15.2%)
Birch pollen allergy	5 (15.2%)
The severity of asthma, if present (mild/moderate/severe)	5 (15.2%)

The data are quoted as the number of physicians (percentage). AIT, allergen immunotherapy; AR, allergic rhinitis.

### Characteristics of the patient population

On average, each physician saw 332 patients a month (range: 180–500). Of these, 209 patients (63%) were consulting for a respiratory allergy. 108 of the 209 (51.7%) received AIT, and a SLIT liquid formulation was prescribed in 86 cases (80.2%).

The 33 participating physicians filled out CRFs (number per physician: 3 to 20) for a total of 414 allergic patients with respiratory allergies. 392 of the 414 CRFs met the study criteria and were analyzed. 258 of the 392 CRFs (65.8%) concerned patients having received a dose adjustment. The characteristics of the 392 patients are summarized in Table 3.

**Table 3:** Characteristics of the patient population.

	Total (*n* = 392)	Dose adjustment (*n* = 258)	No dose adjustment (*n* = 134)	*P*–value
Age (years)				0.581
mean ± SD	27.8 ± 17.5	28.2 ± 18	27.2 ± 16.3	
median [IQR]	23 [13–40]	23.5 [13–40]	23 [14–40]	
range	5–82	5–82	6–74	
Age groups (years)				0.396
≥18	227 (57.9%)	149 (57.8%)	78 (58.2%)	
12–17	94 (24%)	58 (22.5%)	36 (26.9%)	
5–11	71 (18.1%)	51 (19.8%)	20 (14.9%)	
Sex				0.095
Female	197 (50.3%)	138 (53.5%)	59 (44%)	
Male	195 (49.7%)	120 (46.5%)	75 (56%)	
Duration of allergy (years)				0.915
mean ± SD	9.6 ± 7.4	9.5 ± 7.4	9.6 ± 7.4	
median [IQR]	7 [5–11.2]	7 [4–12]	7 [5–10]	
Range	2–40	2–40	2–40	
Number of allergy consultations a year				0.144
mean ± SD	2.5 ± 1.2	2.6 ± 1.2	2.4 ± 1.2	
median [IQR]	2 [2–3]	2 [2–3]	2 [2–3]	
range	1–12	1–12	1–10	
Diseases present upon treatment initiation				
Allergic rhinitis	383 (97.7%)	252 (97.7%)	131 (97.8%)	>0.999
Allergic asthma	128 (32.7%)	86 (33.3%)	42 (31.3%)	0.776
Conjunctivitis	163 (41.6%)	111 (43%)	52 (38.8%)	0.487
Skin manifestations	26 (6.6%)	19 (7.4%)	7 (5.2%)	0.553
Disease-inducing allergens				
House dust mites	150 (38.3%)			
Grass pollen	48 (12.2%)			
Birch pollen	31 (7.9%)			
House dust mites and grass pollen	24 (6.1%)			
Birch pollen and grass pollen	17 (4.3%)			
Cat dander	16 (4.1%)			
Cypress pollen	16 (4.1%)			
House dust mites and cat dander	12 (3.1%)			
House dust mites and birch pollen	8 (2.0%)			
Other allergens or combinations	70 (17.9%)			
ARIA classification (*n* = 383 patients with allergic rhinitis)				
Mild intermittent	10 (2.6%)	7 (2.8%)	3 (2.3%)	
Mild persistent	26 (6.8%)	15 (6.0%)	11 (8.4%)	
Moderate-to-severe intermittent	14 (3.7%)	7 (2.8%)	7 (5.3%)	
Moderate-to-severe persistent	312 (81.5%)	208 (82.5%)	104 (79.4%)	0.593
PAREO score (*n* = 383 patients with allergic rhinitis)				
mean ± SD	7.1 ± 1.7	7.2 ± 1.7	7 ± 1.6	0.337
median [IQR]	7 [6–8]	7[6–8]	7[6–8]	
Range	2–10	2–10	2–10	
GINA stage (*n* = 128 patients with allergic asthma)				
GINA 1	35 (27.8)	22 (26.2)	13 (31.0)	0.926
GINA 2	45 (35.7)	30 (35.7)	15 (35.7)	
GINA 3	40 (31.7)	28 (33.3)	12 (28.6)	
GINA 4	5 (4.0)	3 (3.6)	2 (4.8)	
GINA 5	1 (0.8)	1 (1.2)	0 (0)	
Missing data	2	2	0	

The patient population was relatively young (mean ± SD age on inclusion: 27.8 ± 17.5), and 42.1% were under the age of 18. As expected for a patient population consulting a specialist, the allergic disease was often severe and burdensome (sleep disrupted in 44% of patients, social (70%), scholar and professional activities (63%) disrupted in the large majority of patients): 121 of the 392 patients (30.9%) were poly-allergic, and 312 of the 383 patients (81.5%) with AR on inclusion had moderate-to-severe persistent disease (according to the ARIA classification).

Symptom severity (80%), patient motivation (54%), poorly effective or unwanted symptomatic medications (50%), AR aggravation prevention (41%), asthma presence (31%) or asthma or new sensitization prevention (30%) were the main reasons for suggesting SLIT.

The SLIT treatment coverage (i.e., the proportion of patients with a particular allergy treated with SLIT) differed from one allergen to another, with values of 93% for house dust mites (HDMs), 75% for grass pollen, 78% for birch pollen, and 51% for cat dander. Most of the patients (86%) were receiving one course of SLIT, whereas respectively 13.3% and 0.8% were receiving two or three courses of SLIT simultaneously. More than half of the patients (54.6%) had been treated for 2 years, with 30.4% treated for 3 years, 8.2% treated for 4 years, 4.8% treated for 5 years, and 2% treated for more than 5 years.

Treatment protocol was perennial in 90% of cases for HDM, pre-co seasonal in 88% of cases for grasses, and in 75% of cases for birch pollens.

### Practice with regard to SLIT dose adjustment

**Figure 4:**
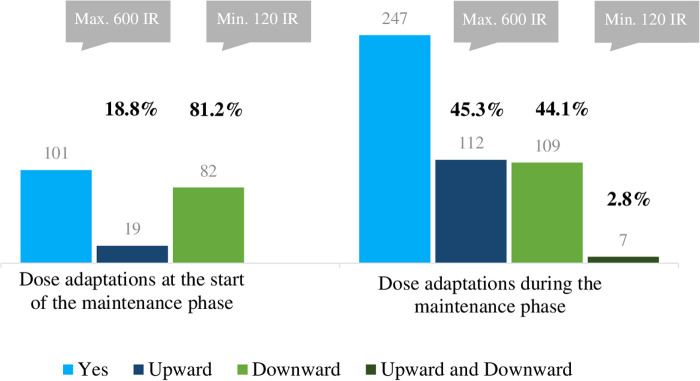
Number of patients with dose adaptation (total *n* = 258) and types of dose adjustment during SLIT, as recorded in the CRF.

Dose adjustments were noted for 65.8% (258 of 392) patients which is beyond the expected proportion of 50% (Refer to section 2.4). Of these patients, 39.2% had a dose adjustment at the start of the maintenance phase (dose reduction: 81.2%; minimum dose: 120 IR / 2 actuations) and 95.7% had an adjustment during the maintenance phase (up and down to a similar extent; max. 600 IR / 10 actuations, min. 120 IR / 2 actuations) (Figure 4).

A wish for greater efficacy was the most common reported reason for dose increases. In contrast, the occurrence of adverse effects, worsening of symptoms, the occurrence of the pollen season and the patient's wishes were the main reasons for dose reductions. Dose adjustment was not associated with the number of allergens received, the patient's sex, or the planned duration of SLIT. Dose reductions (less than 5 actuations per day) were most frequent in treatment year 1 and typically lasted for 1 to 3 months. Dose increase (more than 5 actuations per day) was most frequent in treatment year 2 and lasted until the end of the treatment.

For the study population as a whole (*n* = 392), 53.6%, 25.8%, 15.3%, and 8.7% of the patients were being treated with HDM, grass pollen, birch pollen, and cypress pollen SLIT, respectively.

When considering each type of allergen extract, most of the dose adjustments for patients treated with cat dander extract or *Alternaria* extract were dose increases, both at the start of the maintenance phase and during the maintenance phase. The maximum observed dose was 220 IR / 11 actuations per day. In contrast, dose reductions (at the start of the maintenance phase and during the maintenance phase) were significantly more frequent in patients treated with birch pollen SLIT.

61% of the physicians considered that dose increases were associated with greater effectiveness, and 85% considered that dose reductions were associated with greater safety.

### Effectiveness of SLIT

**Figure 5:**
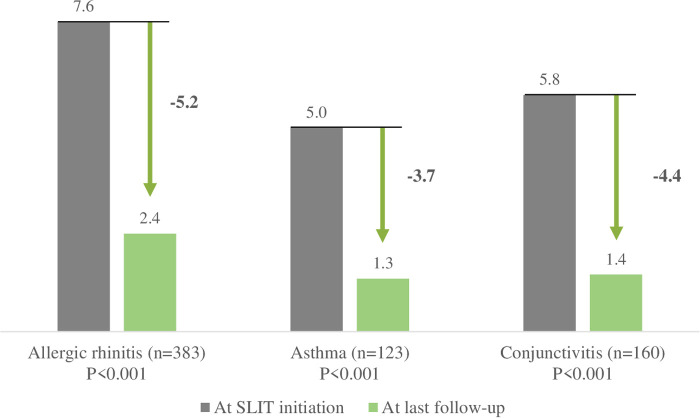
Changes in symptom severity during SLIT, as recorded on a VAS in the CRF by the physician.

All measures of disease intensity (on a VAS for symptom severity, quality of life, symptomatic medication use, and the PAREO score) significantly fell during SLIT (Figure 5). The overall mean AR symptom severity (rated by the physician) fell from 7.6 at SLIT initiation to 2.4 at last follow-up (change: 5.2). This score decreased from 7.4 to 2.0 in the subgroup of patients without dose adjustment and from 7.6 to 2.6 in the subgroup with dose adjustment.

The mean asthma symptom severity score fell from 5.0 to 1.3 (change: 3.7) overall, from 4.8 to 1.0 in the subgroup of patients without dose adjustment, and from 5.0 to 1.4 in the subgroup with dose adjustment. The mean conjunctivitis symptom severity score fell from 5.8 to 1.4 overall, from 5.7 to 1.1 in the subgroup of patients without dose adjustment and from 5.8 to 1.5 in the subgroup with dose adjustment. The mean ± SD PAREO score fell significantly (from 7.1 ± 1.7 to 2.6 ± 2.4; mean ± SD change: 4.5 ± 2.4; *p* = 0.006; i.e., the AR became less severe) for the patient population as a whole, from 7.0 ± 1.6 to 2.2 ± 2.2 in the subgroup of patients without dose adjustment, and from 7.2 ± 1.7 to 2.9 ± 2.5 in the subgroup with dose adjustment.

With regard to the impact of allergy symptoms on quality of life, there was a statistically significant change over time; the score fell from 7.5 at SLIT initiation to 2 at last follow-up (*p* < 0.001). Symptomatic medication use also fell significantly over the course of SLIT. In patients with AR, the use of nasal/oral antihistamines fell from 7.8 at SLIT initiation to 2.1 at last follow-up. Similarly, the use of nasal corticosteroids decreased from 5.8 at SLIT initiation to 1.5 at last follow-up. In patients with GINA step 1 asthma, the use of short-acting beta agonists fell from 3.5 at SLIT initiation to 0.9 at last follow-up. The use of fixed associations for patients with GINA 2 and 3 asthma also fell significantly, from 4.6 and 7.9 at SLIT initiation to 0.9 and 3.1 at last follow-up, respectively. The very few differences in effectiveness and wellbeing between patients with vs. without dose adjustment were not clinically relevant.

## Discussion

Our present results show that precision dosing is anchored in French physicians' clinical practice. Dose adjustment (whether upwards or downwards) is frequent at the start of the maintenance phase and during the maintenance phase of SLIT in patients treated with STALORAL® by specialist physicians in France.

A secondary objective of this study was to evaluate the clinical benefits of a treatment course with SLIT as assessed by the physicians. The results showed that SLIT has a real, clinically relevant impact on allergy symptoms. As mentioned above, the mean AR symptom severity fell from 7.6 at SLIT initiation to 2.4 at last follow-up. The change of 5.2 is much greater that the value of 2.3 quoted by Demoly et al. as a clinically relevant variation in quality of life and symptoms in patients treated for AR in primary care (35) and the value of 2.0 quoted by Bousquet et al. as a step-down in disease severity again among patients treated for AR in primary care (36).

It is noteworthy that equivalent clinical results were obtained with the recommended standard dose of SLIT and doses greater or lower than standard dose—suggesting that the allergy specialist was able to modulate the SLIT dose according to the patient's profile and/or response to treatment (effectiveness and tolerability). This approach might enable the achievement of individual treatment targets defined upon initiation of AIT, such as a reduction in symptoms or in medication use, or better quality of life.

The present study had a number of strengths. Firstly, MaDo was the first study designed to highlight the need and the reasons for SLIT dose adjustments in France. Secondly, the study population was representative of allergists and other AIT prescribers in France. Thirdly, the response rate (18.9%) was relatively high for this type of study, indicating a particular interest in this topic amongst the invited physicians. Lastly, the proportion of case reports (65.8%) exceeded the 50% estimated for sample size calculation enabling the study to reach its objectives. The study had some limitations, some of which were related to its retrospective design. Firstly, there may have been selection bias; it is likely that the physicians who agreed to participate in the study were more likely to employ dose adjustment than those who did not. Secondly, the results were solely representative of the healthcare system in France and cannot necessarily be extended to other countries or systems. Thirdly, the number of participating physicians was quite small (*n* = 33) with a majority of allergists working in private practice. No pediatricians, ENT specialists or internal medicine specialists participated. Nevertheless, as mentioned above, the physician population was representative of a reference group of AIT-prescribing specialist physicians (*n* = 434). Finally, more information on safety data would have provided additional insights into the overall benefit-risk assessment of dose adjustment (whether upwards or downwards) notably when efficacy outcomes are equivalent.

In conclusion, dose adjustment of SLIT liquid preparations as a function of the patient profile and/or treatment response is frequent throughout the first 2 years of therapy and is associated with similar levels of effectiveness (for AR and asthma) to patients receiving recommended dosing. Precision dosing might optimize the overall benefit-risk profile of AIT for individual patients throughout their entire treatment course, enabling them to achieve both short- and long-term treatment goals, whilst maximizing the safety and tolerability.

## Data Availability

The original contributions presented in the study are included in the article/Supplementary Material, further inquiries can be directed to the corresponding author/s.

## Footnotes

1 PAREO (Prurit/nasal pruritus, Anosmie/anosmia, Rhinorrhée/rhinorrhea, Eternuements/sneezing, Obstruction nasale/nasal obstruction).
